# Executive Processes Underpin the Bilingual Advantage on Phonemic Fluency: Evidence From Analyses of Switching and Clustering

**DOI:** 10.3389/fpsyg.2019.01355

**Published:** 2019-06-12

**Authors:** John E. Marsh, Patrik Hansson, Daniel Eriksson Sörman, Jessica Körning Ljungberg

**Affiliations:** ^1^Department of Environmental Psychology, University of Gävle, Gävle, Sweden; ^2^School of Psychology, University of Central Lancashire, Preston, United Kingdom; ^3^Department of Psychology, Umeå University, Umeå, Sweden; ^4^Division of Human Work Science, Luleå University of Technology, Luleå, Sweden

**Keywords:** bilingualism, aging, phonemic fluency, executive function, longitudinal study

## Abstract

Bilinguals often show a disadvantage in lexical access on verbal fluency tasks wherein the criteria require the production of words from semantic categories. However, the pattern is more heterogeneous for letter (phonemic) fluency wherein the task is to produce words beginning with a given letter. Here, bilinguals often outperform monolinguals. One explanation for this is that phonemic fluency, as compared with semantic fluency, is more greatly underpinned by executive processes and that bilinguals exhibit better performance on phonemic fluency due to better executive functions. In this study, we re-analyzed phonemic fluency data from the Betula study, scoring outputs according to two measures that purportedly reflect executive processes: clustering and switching. Consistent with the notion that bilinguals have superior executive processes and that these can be used to offset a bilingual disadvantage in verbal fluency, bilinguals (35–65 years at baseline) demonstrated greater switching and clustering throughout the 15-year study period.

## Introduction

The ability to communicate in more than one language—bilingualism or multilingualism —is common worldwide, and rapidly increasing (Moreno and Kutas, 2005; Bhatia and Ritchie, 2013). For example, globally, approximately two thirds of children are raised as bilingual (Associated Press, 2001, as cited in Marian and Shook, 2012). The existence of bilingualism offers a window through which the mechanisms of language production can be studied, particularly in relation to the speed of access of words, depending on which language (first/dominant or second language) is tapped (e.g., Segalowitz and Hulstijn, 2005). Despite the apparent additional load of possessing another language, proficient bilinguals appear to make daily use of both languages in a competent and effortless manner. However, there is some evidence that speaking more than one language entails a cost for language production that is manifest in prolonged reaction time and lower accuracy in lexical access tasks such as picture naming. This is referred to as the *bilingual disadvantage* (e.g., Gollan et al., 2007; Ivanova and Costa, 2008; Shao et al., 2014) and is thought to be a result of a between-language interference among bilinguals (Sandoval et al., 2010).

Contrary to this, a body of additional evidence suggests that speaking more than one language is associated with better metalinguistic knowledge, i.e., the capability of reflecting and evaluating language and focusing, and directing, attention to particular language aspects (Galambos and Goldin-Meadow, 1990; Campbell and Sais, 1995; Bialystok, 1998; Bialystok et al., 2003; Adesope et al., 2010). Moreover, bilinguals have been shown to outperform monolinguals on tasks tapping executive functions such as attentional control (Galambos and Goldin-Meadow, 1990; Bialystok, 1999, 2006, 2007, 2009; Bialystok and Viswanathan, 2009), inhibition (Bialystok et al., 2004, 2009; Cottini et al., 2015) and switching (Bialystok et al., 2008a; Martin-Rhee and Bialystok, 2008). This superior performance for bilinguals over monolinguals has been coined the *bilingual advantage*. The current study sought to determine whether co-opting executive processes—measurable through switching and clustering scores—in the context of a language production task, phonemic fluency (Troyer et al., 1997), reduces the more typically observed bilingual disadvantage in lexical access (e.g., Gollan et al., 2005b; Bialystok et al., 2008b; Ivanova and Costa, 2008; Sandoval et al., 2010), or instead leads to a bilingual advantage. Such analytic scrutiny may shed light on why a bilingual advantage in phonemic fluency is sometime found (Luo et al., 2010; Ljungberg et al., 2013).

By now, it is reasonably well established that bilingualism slows down or otherwise impairs lexical access: Bilinguals, compared to monolinguals, are typically slower at picture naming (Gollan et al., 2005b), or name fewer pictures from standardized sets (e.g., Boston Naming Test; Roberts et al., 2002; Gollan et al., 2005b, 2007; Bialystok et al., 2008b; Tao et al., 2015). Moreover, bilinguals correctly identify fewer words in noise (Rogers et al., 2006) and exhibit greater so-called tip-of-the-tongue (TOT) retrieval states that occur when individuals have the phenomenological experience of being on the verge of, but temporarily unable, to access information in long-term memory (Gollan and Silverberg, 2001; Gollan and Acenas, 2004; Gollan et al., 2005a).

One commonly used language production task that has yielded arguably the most convincing evidence for a bilingual disadvantage is that of verbal fluency, a short test of verbal functioning (e.g., Rosselli et al., 2000; Gollan et al., 2002; Portocarrero et al., 2007; but see Obler et al., 1986; Bialystok et al., 2008b). In a verbal fluency task, the participant is given a short time (usually 60 s) to generate as many members of a semantic category (e.g., “four-legged animals”) or letter/phonemic category (e.g., “words that begin with *f*”) as possible (Benton, 1968; Newcombe, 1969; Jones et al., 2012; Marsh et al., 2017). Usually, the responses are oral, but output can also be written (e.g., Jones et al., 2012). Bilinguals typically produce fewer responses than monolinguals regardless of whether testing is restricted to their first-learned (Ransdell and Fischler, 1987; Ivanova and Costa, 2008), or dominant, language (Gollan and Acenas, 2004; Gollan et al., 2005b). However, the bilingual disadvantage in the context of verbal fluency is often qualified by the nature of the verbal fluency task, with the effect being stronger, or more readily observed in semantic, as compared with, phonemic fluency. This is typically attributed to greater cross-language interference in semantic as opposed to phonemic fluency (Rosselli et al., 2000; Gollan et al., 2002; Portocarrero et al., 2007; but see Luo et al., 2010, for a possible mediating role of vocabulary size; see also Vega-Mendoza et al., 2015). Moreover, several studies have shown a bilingual advantage in phonemic fluency (e.g., Sandoval et al., 2010) which has been attributed to compensation mechanisms related to executive control since phonemic fluency, as compared with semantic fluency, requires more by way of these (Hilchey and Klein, 2011).

Episodic and Working Memory processes—including those related to executive function—play several roles in verbal fluency (cf. Graesser and Mandler, 1978; Henry and Crawford, 2004). Such processes are likely involved in monitoring the output of words from memory to ensure they are consistent with the task constraints and not a repetition (perseveration) of a previous item (Rosen and Engle, 1997; Fisk and Sharp, 2004). For example, in the case of phonemic fluency participants must avoid producing “phonemic parallels” – letters that begin with the same onset phoneme as legitimate responses (e.g., pharmacy) but are illegitimate responses in the context of the specific cue (e.g., produce words that “begin with f”; see Jones et al., 2012; Experiment 5). Therefore, task set (instructions for generation, including exclusion criteria) and prior responses must be retained in Working Memory, and irrelevant responses and repetitions must be suppressed (Hirshorn and Thompson-Schill, 2006). Breakdown of, or deficits in, verbal ability or executive processes responsible for suppression, result in poor fluency performance, thereby substantiating the use of verbal fluency tasks as instruments that can be used to rapidly, and reliably, screen for changes in general verbal and executive function (Roca et al., 2012).

The current study focuses only on phonemic fluency but here we contrast, across other studies, this form of language production with semantic fluency to illustrate the processes that are involved in the tasks and the explanations for why they are associated with a bilingual disadvantage/advantage. While both tasks are thought to tap executive control (Henry and Crawford, 2004; Fitzpatrick et al., 2013) it is not surprising that semantic and phonemic fluency are thought to be underpinned by both similar and dissimilar processes. For example, phonemic fluency relies heavily on phonological or lexical retrieval mechanisms (e.g., Levelt et al., 1999), whereas semantic fluency requires access to semantic knowledge (Butters et al., 1987). Moreover, phonemic, but not semantic fluency, can be supported by phonemic and orthographic cues (Bokat and Goldberg, 2003). Furthermore, it has been argued (Gruenewald and Lockhead, 1980; Wixted and Rohrer, 1994; Troyer et al., 1997; Rosselli et al., 2000; Gollan et al., 2002), that verbal fluency is underpinned by multiple processes that are not captured by the total number of words produced in the allotted time. As ‘process’ approaches in neuropsychological testing hold, the strategies used by individuals to perform a particular task should be examined in addition to overall performance level (Abwender et al., 2001).

In the context of processes underpinning verbal fluency, Troyer et al. (1997) distinguish clustering and switching. In the context of phonemic fluency, clustering refers to the process of producing successive words that are phonologically related (e.g., words beginning with the same first two letters “fa”), words that rhyme (“fog,” “flog”) or homonyms (e.g., sail and sale) to one another. Such words are clustered together during output and cluster size refers to the mean number of successively presented words in each clusters. Conversely, switching refers to the process of changing subcategories by altering search criteria, or producing words that do not belong to clusters (Troyer et al., 1997), thus the sequence “farce, farm, fast, fog, flog, and frog” has two clusters and one switch (from a cluster wherein words begin with the same two first letters to a cluster of rhyming words). Analysis of clustering and switching processes occurring within verbal fluency trials enables a more qualitative, in-depth analysis of verbal fluency performance to be conducted.

Phonemic fluency involves a somewhat unusual mode of lexical retrieval, underpinned by processes that are rarely used in everyday speech production (Rosen, 1980). Unlike semantic fluency, responses are not based on concepts that are clustered along semantic properties that can be used as a retrieval aid to automatically access responses. Phonemic fluency requires retrieval of lexical entries that are not typically organized alphabetically. Therefore no existing structure exists to support their recall (Strauss et al., 2006). Furthermore, phonemic fluency often entails production of function words, words of different classes and abstract words, as opposed to nouns/concrete words that semantic fluency requires (Rosselli et al., 2000). The links between same-first-letter words are likely to be weaker than for same-category words (Shao et al., 2014) and phonemic as compared with semantic fluency may require search within more semantic categories (Filippetti and Allegri, 2011). In this way, it is generally argued that phonemic, as compared to semantic, fluency is more strictly undergirded by novel, strategic search processes and controlled processing/executive function, than the automatic activation of words (Troyer et al., 1997; Portocarrero et al., 2007; Filippetti and Allegri, 2011; Sauzéon et al., 2011; Friesen et al., 2015). Such executive processes may be involved in the suppression of the habitual use of words according to their meaning (Perret, 1974) including the suppression of the activations of semantically associated words (e.g., Luo et al., 2010; Katzev et al., 2013). Moreover, participants must exclude proper nouns, repetitions and variants of the same words with different endings (morphological variants) such as “ache” and “aching” (Troyer et al., 1997). Even though there are potentially a lot more responses for a letter as opposed to semantic categories, phonemic fluency is generally thought of as the more difficult, and effortful, task with fewer items generated than for semantic fluency (Gollan et al., 2002; but see Azuma et al., 1997). Behavioral (e.g., divided attention; Troyer et al., 1997) and neuropsychological (Troyer et al., 1998) evidence supports the assertion that subcomponents of phonemic fluency, such as switching, are underpinned by executive processing. For example, Troyer et al. (1998) showed that patients with focal frontal lobe lesions of the prefrontal cortex areas associated with executive control performed poorer than controls on subcomponent measures of the phonemic fluency task.

One contributing reason as to why a bilingual advantage sometimes manifests for phonemic fluency is that the task is relatively immune from cross-language interference that may contribute to the bilingual disadvantage in semantic phonemic fluency tasks. In the context of the phonemic fluency task, if the task involves generation of “d” words, the translation of “dog” (English) to “hund” (Swedish) can easily be suppressed because it does not start with the same first letter (the target letter; Kormi-Nouri et al., 2012). In the context of semantic fluency, this poses a problem because the translation equivalents (“dog,” “hund”) may come to mind during retrieval and require suppression. Indeed, intrusion of translation equivalents are taken as evidence of cross-language interference (Sandoval et al., 2010). Moreover, phonemic fluency involves retrieval of more abstract than concrete words which are more difficult to automatically translate and therefore produce less interference than concrete words (e.g., Tokowicz et al., 2002). Of course, one must be careful here not to give the impression that semantic fluency requires more executive control than phonemic fluency. While intrusions in semantic fluency may represent failures of executive functions such as monitoring and suppression, the burden on executive processing in strategic search—e.g., not according to semantic criteria—in the context of phonemic, as compared with phonemic, fluency is overall much greater (Troyer et al., 1997).

Counterintuitively, the same mechanism that causes a bilingual disadvantage—interference between languages—may be responsible for the development of stronger executive control in bilinguals as compared with monolinguals (Bialystok et al., 2009; Kroll et al., 2012; but see Sandoval et al., 2010; Gollan et al., 2011).

As detailed in the foregoing, phonemic fluency performance is widely held to be characterized by executive processes. The impetus of the current study was fueled by the notion that phonemic fluency is an executively laden task that comprises readily computed sub-processes (switching and clustering). The contribution of these executive processes to phonemic fluency have not yet been fully explored. For example, in Ljungberg et al. (2013), which was based on data from the Betula longitudinal study (Nilsson et al., 1997, 2004), it was shown that bilingual participants outperformed monolinguals in phonemic fluency. However, only the number of words that the participants generated were considered and possible processes that underpin the bilingual advantage in phonemic fluency performance were not considered. The current study aimed to investigate the contribution of executive control mechanisms to supporting phonemic fluency, thereby offering some clarity as to why a bilingual advantage is sometimes manifest in the phonemic fluency task (e.g., Ljungberg et al., 2013). Cohering with prior analysis of phonemic fluency protocols (Troyer et al., 1997), the use of switching and clustering indices—measures of executive processing—were adopted to facilitate a more fine-grained analysis of the processes involved in verbal fluency, and a potential difference between monolinguals and bilinguals in language production (for alternative, computational methods for analyzing bilingual verbal fluency performance, see Hills et al., 2012; Taler et al., 2013).

As in Ljungberg et al. (2013), data in the current study were examined from the Betula longitudinal study. The Betula longitudinal study is an unparalleled, large scale study permitting access to bilingual and monolingual participants in numbers not typically observed in verbal fluency experiments (e.g., Troyer et al., 1997) or bilingual studies generally. This study also allows the investigation of the relationship between bilingualism and phonemic fluency (and executive processes used therein) over a follow-up period of 15 years. Such longitudinal studies are very rare in the literature, and the Betula longitudinal study allows for greater control over potentially confounding variables. For example, bilinguals and monolinguals share the same native language (Swedish) as opposed to differing native languages. This unique aspect reduces the risk of variance in performance due to smaller vocabulary in one language for the bilinguals (Sörman et al., 2017). Therefore, finding a bilingual advantage in phonemic fluency within the current study is significantly less likely to be due to differences in vocabulary size between bilinguals and monolinguals. This was the conclusion reached by Luo et al. (2010) who found that bilingual advantages in letter fluency could be seen for bilinguals high in vocabulary size compared to monolinguals and bilinguals low in vocabulary size and attributed this to their greater executive function. In our study, differences in vocabulary size between monolinguals and bilinguals are mitigated because bilinguals are at least assumed to have large vocabulary size since they are randomly sampled with the criteria that Swedish is their first language and that they live in a country wherein only their first language is used in the society. Therefore, it would be reasonable to assume that any bilingual advantage reported in the current study would have to do more with enhanced cognitive control than vocabulary size *per se*.

Since they are a purported index of executive processing that may be enhanced in bilinguals (e.g., Bialystok et al., 2008a), it was hypothesized that switching and clustering measures would be greater for bilinguals as compared to monolinguals and that this difference would be evident across the follow-up test periods. Moreover, given that age has a small or minimal effect size on switching and clustering in the context of phonemic fluency (Troyer et al., 1997), we did not expect differences within groups across follow-up test periods. Generally, these results would cohere nicely with the notion that the executive functions that bilinguals practice through everyday language switching can positively affect phonemic fluency and thus offset the bilingual disadvantage that is observed with other language production tasks such as semantic fluency (Gollan et al., 2002; Bialystok et al., 2008b; cf. Luo et al., 2010).

## Materials and Methods

### Participants

The participants were drawn from the Betula Prospective Cohort Study of aging, memory, and dementia (Nilsson et al., 1997, 2004). The Betula study is a longitudinal study conducted in the northern part of Sweden and been going on for over 25 years. The main objectives were to study memory functions during adult life and old age. Data has been collected at six test waves; 1988–1990 (T1), 1993–1995 (T2), 1998–2000 (T3), 2003–2005 (T4), 2008–2010 (T5), and 2013–2015 (T6). At T1 participants were selected based on a stratified random sampling strategy divided into ten age cohorts; 35, 40, 45, 50, 55, 60, 65, 70, 75, and 80 years. Each cohort consisted of 100 persons and the total number of trial participants (Sample 1) at the first test round was thus 1,000. All participants were screened for dementia; sensory impairments, and a native tongue other than Swedish (see Nilsson et al., 1997 for further details concerning recruitment and inclusion criteria). An advantage of using this sample was that various factors that could be important in assessing bilingual cognitive functions were controlled. These include proficiency in a language, socioeconomic status, sociolinguistic variables (if two languages simultaneously, or just one, are readily used within the community within which one is immersed), patterns of language use, cultural influences and typological similarity between the two languages spoken by the bilingual (Tao et al., 2015). Moreover, all participants were tested in the same language, their mother tongue. It is worth noting here that participants in the Betula study self-report their language proficiency. This is common within studies addressing potential differences in cognitive function between bilinguals and monolinguals and numerous studies have confirmed the relation between self-reported measures and objective measures in tasks such as picture naming (Gollan et al., 2012; Sheng et al., 2014).

For the present study, a total sample of 197 participants (51% women) were included at base line (because of limitation of cluster/switching data, we only included data from Test wave 1 to 4 for Sample 1). The age at baseline ranged from 35 to 65 years (*M* = 52.4, *SD* = 9.2). Table 1 provides demographic data for monolingual (*n* = 139) and bilingual (*n* = 58) participants. This study was approved by the Ethics Review Board, Umeå University, and all subjects gave written informed consent in accordance with the Declaration of Helsinki.

**Table 1:** Baseline characteristics of monolingual and bilingual participants, respectively.

	Monolinguals		Bilinguals	
	Mean	*SD*	Mean	*SD*
Age	55.9	7.0	44.1	8.3^∗∗^
Female sex, %	50.0		55.2	
Education, years	8.0	2.4	16.1	3.6^∗∗^
MMSE	28.1	1.4	28.6	1.4^∗^
Block design	26.9	8.7	33.8	8.2^∗∗^
SRB	19.7	4.9	25.5	2.5^∗∗^
*n*	139		58	

*MMSE, Mini Mental State Examination; SRB, 30-item vocabulary test. ^∗^p < 0.05, ^∗∗^p < 0.001*.

At each test wave, data was collected at two sessions, about 1 week apart. Each test session lasted around 1.5–2 h, in which the first included a health examination and questionnaires, and the second comprised an extensive battery of cognitive assessment. All participants signed a written informed consent and during each test session participants were requested to use glasses or hearing aids if normally used, and they were all tested individually.

### Measures

#### Verbal Fluency and Number of Switches Between Clusters

The participants was required to generate as many words as possible, except personal names, with an initial letter A during 1 min (Rönnlund and Nilsson, 2006). Errors and repetition were not included in the total word count. Definition of a cluster, calculation of cluster size, and the number of switches between clusters (the dependent variable) was in accordance with Troyer et al. (1997) in the following way: Clusters were two or more adjacent words beginning with the same two letter (art and ark), rhymed (arm and alarm), or were homonyms (ale and ail). For the computation of clustering, repetitions and errors were included as these provide detail concerning the course of lexical retrieval during the verbal fluency trials (this followed the procedure of Troyer et al., 1997). Two different measures were calculated from the cluster scoring, these were cluster size and cluster number. Switches were scored as changes between clusters and individual non-clustered words.

#### Inter-Rater Reliability

Coding was performed by one primary and one secondary rater following the detailed procedure for scoring cluster size and switches (Troyer et al., 1997). The scoring procedure is straightforward and unambiguous and this was reflected by the negligible proportion of disagreements between coders (less than 0.05% of all categorizations) that were resolved through discussion between the primary and secondary raters. Due to the small number of disagreements, inter-rater reliabilities were not computed. However, the agreements were clearly consistent with the inter-rater reliabilities (e.g., *r* > 0.95) typically reported using the scoring procedure (Troyer et al., 1997).

#### Language History Questionnaire and Vocabulary

As is typical in many studies of bilingual cognitive function (Lehtonen et al., 2018), we used a self-report questionnaire concerning participants’ ability to speak a second language. If participants indicated that they spoke a second language then they completed questions ranging from 1 (very poor) to 6 (excellent) about their ability to read, write, speak, and listen to a second language. Participants with a score of 4 and higher across all abilities were categorized as bilinguals, while participants using only one language (i.e., Swedish) were categorized as monolinguals, a procedure previously used (see Ljungberg et al., 2013). The majority (95%) of the bilinguals in this study reported English as their second language; they began to learn English in primary school (at the age of 9), and had approximately 7 years of formal training. 93% of the participants indicated that they mainly used their second language “when traveling” or “at work,” and 7% mainly “at home.” Approximately 80% of the bilingual participants indicated that they spent between 0 and 2 h a day reading, writing, speaking, and listening in their second language. Additionally, the participants undertook a vocabulary test which was a 30-item, multiple-choice, synonym test (SRB: Rönnlund and Nilsson, 2006). The task was to select a synonym of the target word from among five alternatives. Seven minutes were allotted for test completion.

#### Covariates

Education was defined by years of formal schooling at baseline. Age was defined by the chronological age of the participants at baseline. In additional *post hoc* analyses we used Mini mental state examination (MMSE; Folstein et al., 1975) and WAIS-R Block Design test (Wechsler, 1981) to control for the participants’ global cognitive status and visuospatial ability (fluid ability) at baseline. Performance on the Block design test has been shown to correlate to a great extent with measures of general intelligence (Ryan et al., 1990).

### Statistical Analysis

An appropriate way to analyze psychological change is with Structural Equation Modeling (SEM). SEM enable analyses of variables on latent level and dependencies among psychological constructs without measuring errors (Nachtigall et al., 2003). We used Latent Growth Modeling (LGM) to investigate whether, if any, time-related changes (across four test occasions stretching over 15 years) in cluster switching, number of clusters, and cluster size were associated with bilingualism, adjusted for age and education. In further *post hoc* analyses we also control for global cognitive status (using MMSE) and fluid ability (using the Block design test). Data were analyzed with SPSS-25 and Amos-25 using full information maximum likelihood (FIML) estimation.

**FIGURE 1:**
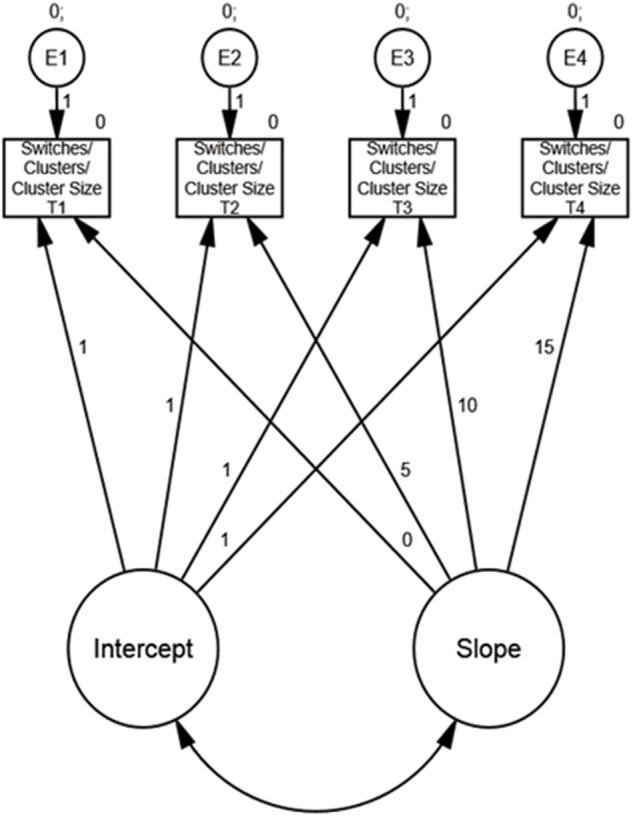
Unconditional LGM. Numbers show *a priori* regression weights.

First, the fit of unconditional LGM’s (see Figure 1) including four time points of cluster switching, number of clusters, and cluster size were estimated. Of relevance for further analyses, these models inform of mean and variance in intercept and slope.

Next, we fitted conditional LGM’s where the intercept and slope were regressed on language ability (bilingual = 1 and monolingual = 0) and covariates (see above). Bentler’s comparative fit index (CFI), the root-mean-square error of approximation (RMSEA), and Chi-square/df was used to explore the fit of each model. A CFI greater than ≥ 0.95 is warranted for acceptable fit, and for RMSEA a value of 0.06 or less is indicative of acceptable model (Hu and Bentler, 1999). Suggested upper thresholds for normed Chi-square differ between 2.0 (Tabachnick and Fidell, 2007) to 5.0 (Wheaton et al., 1977) in the literature. Estimates (regression weights) were used to explore the association between covariates and baseline performance (the intercept) and time-related change of performance (the slope).

Since our main concern was to investigate the differences in executive processes used in verbal fluency, the measure of phonemic fluency performance in terms of number of words generated will only be considered for descriptive reasons in the results section below. The results from modeling that variable will be presented in Appendix 1 because of the redundancy with the results obtained in Ljungberg et al. (2013).

## Results

Table 2 presents descriptive statistics for the dependent variables across the four test waves and for monolingual and bilingual participants, respectively.

**Table 2:** Descriptive statistics for the dependent variables across monolingual and bilingual participants and test waves, respectively.

	Monolinguals			Bilinguals		
	
	Mean	*SD*	*N*	Mean	*SD*	*N*
**Switches**						
T1	6.43	4.40	139	8.74	3.61	58
T2	6.80	3.73	133	10.32	3.04	55
T3	6.32	2.94	133	9.90	3.95	57
T4	5.99	3.10	116	9.26	3.76	39
**Clusters**						
T1	1.40	1.20	139	2.69	1.52	58
T2	1.50	1.23	133	2.69	1.66	55
T3	1.68	1.24	133	2.47	1.35	57
T4	1.28	1.08	116	2.10	1.20	39
**Cluster Size**						
T1	2.43	2.48	139	4.62	3.29	58
T2	2.50	2.52	133	3.83	2.79	55
T3	2.56	2.58	133	3.65	3.97	57
T4	2.05	2.02	116	3.26	2.49	39

Table 3 presents correlations between the measures at baseline. As can be seen, and expected, age correlated negatively with all other measures (except sex). Besides age, almost all measures were positively related to each other indicating, for example, that more years of formal schooling were associated with better performance on both switching (*r* = 0.28, *p* < 0.001), number of clusters (*r* = 0.40, *p* < 0.001), and cluster size (*r* = 0.32, *p* < 0.001). This pattern also holds for bilingualism (coded 1 = bilingual, 0 = monolingual) where the correlation was *r_pb_* = 0.29, *p* < 0.001 with switching performance, *r_pb_* = 0.41, *p* < 0.001 with number of clusters, *r_pb_* = 0.34, *p* < 0.001 with cluster size, and *r_pb_* = 43, *p* < 0.001 with number of words. Sex did not have any relation with the other variables, see Table 3, and will therefore be discarded in following analyses.

**Table 3:** Person’s (point-biserial when dichotomous variabels) correlation between variabels at baseline.

	1	2	3	4	5	6	7	8	9	10
(1) Age	—										
(2) Education	–0.58 ^∗∗∗^	—									
(3) Sex	0.01	–0.03	—								
(4) ^∧^Bilingual	–0.59 ^∗∗∗^	0.79 ^∗∗∗^	–0.05	—							
(5) Words	–0.28 ^∗∗∗^	0.41 ^∗∗∗^	0.09	0.43 ^∗∗∗^	—						
(6) Switches	–0.19 ^∗∗^	0.28 ^∗∗∗^	0.05	0.29 ^∗∗∗^	0.79 ^∗∗∗^	—					
(7) Clusters	–0.31 ^∗∗∗^	0.40 ^∗∗∗^	0.01	0.41 ^∗∗∗^	0.69 ^∗∗∗^	0.30 ^∗∗∗^	—				
(8) Cluster size	–0.23 ^∗∗^	0.32 ^∗∗∗^	0.09	0.34 ^∗∗∗^	0.63 ^∗∗∗^	0.03	0.76 ^∗∗∗^	—			
(9) MMSE	–0.03	0.23 ^∗∗^	0.01	0.15 ^∗^	0.28 ^∗∗∗^	0.27 ^∗∗∗^	0.25 ^∗∗∗^	0.13	—		
(10) Block design	–0.33 ^∗∗∗^	0.34 ^∗∗∗^	–0.04	0.35 ^∗∗∗^	0.32 ^∗∗∗^	0.26 ^∗∗∗^	0.24 ^∗∗∗^	0.21 ^∗∗^	0.25 ^∗∗∗^	—	

*^∧^bilinguals = 1, monolinguals = 0; MMSE, Mini Mental State Examination; **^∗^**p < 0.05, ^∗∗^p < 0.01, ^∗∗∗^p < 0.001.*

Moving on to the main analyses, the unconditional LGM regarding switches (see Figure 1), the fit indices were: CFI = 0.969, RMSEA = 0.090, and Chi-square/df = 2.547. Means, variances, and covariance for/between performance at baseline (the intercept) and time-related change (the slope) are displayed in Table 4 (Switches). Regarding change (the slope), no significant results (*p* > 0.05) were obtained. This indicates no statistically significant change across the four time points or no significant variation between participants regarding the rate of change. However, a significant variance in the intercept was found, indicating non-trivial variation in baseline performance between the participants.

**Table 4:** Estimates from the Unconditional LGM’s.

	Switches		
**Means**	**Estimate**	***SE***	***P***

I (Intercept)	7.420	0.249	0.001
S (Slope)	–0.160	0.017	0.339
**Variance**			
I (Intercept)	7.860	1.249	0.001
S (Slope)	0.008	0.009	0.347
Covariance of I and S	–0.027	0.860	0.752

	**Clusters**		

**Means**	**Estimate**	***SE***	***P***
I (Intercept)	1.861	0.096	0.001
S (Slope)	–0.013	0.008	0.097
**Variance**			
I (Intercept)	1.060	0.217	0.001
S (Slope)	0.004	0.002	0.016
Covariance of I and S	–0.040	0.016	0.012

	**Cluster size**		

**Means**	**Estimate**	***SE***	***P***
I (Intercept)	3.109	0.192	0.001
S (Slope)	–0.031	0.015	0.045
**Variance**			
I (Intercept)	4.345	0.806	0.001
S (Slope)	0.019	0.006	0.002
Covariance of I and S	–0.040	0.016	0.012

For the unconditional LGM concerning number of clusters, (see Figure 1), the fit indices were: CFI = 0.902, RMSEA = 0.093, and Chi-square/df = 2.695. Means, variances, and covariance for/between performance at baseline (the intercept) and time-related change (the slope) are displayed in Table 4 (Clusters). Regarding change (the slope), only a significant variance (*p* = 0.02) was obtained. This indicates no overall statistically significant change across the four time points but a non-trivial variation between participants regarding the rate of change. Further, a significant variance in the intercept was found, indicating non-trivial variation in baseline performance between the participants.

For the unconditional LGM concerning number of cluster size, (see Figure 1), the fit indices were: CFI = 1.000, RMSEA = 0.000, and Chi-square/df = 0.636. Means, variances, and covariance for/between performance at baseline (the intercept) and time-related change (the slope) are displayed in Table 4 (Cluster size). Regarding change (the slope), there was a significant negative rate of change across time (β = -0.031, *SE* = 0.008, *p* = 0.045) accompanied by a significant variance in the rate of change (*p* = 0.002). This indicates an overall statistically significant change across the four time points and a non-trivial variation between participants regarding the rate of change. Further, a significant variance in the intercept was found, indicating non-trivial variation in baseline performance between the participants.

**FIGURE 2:**
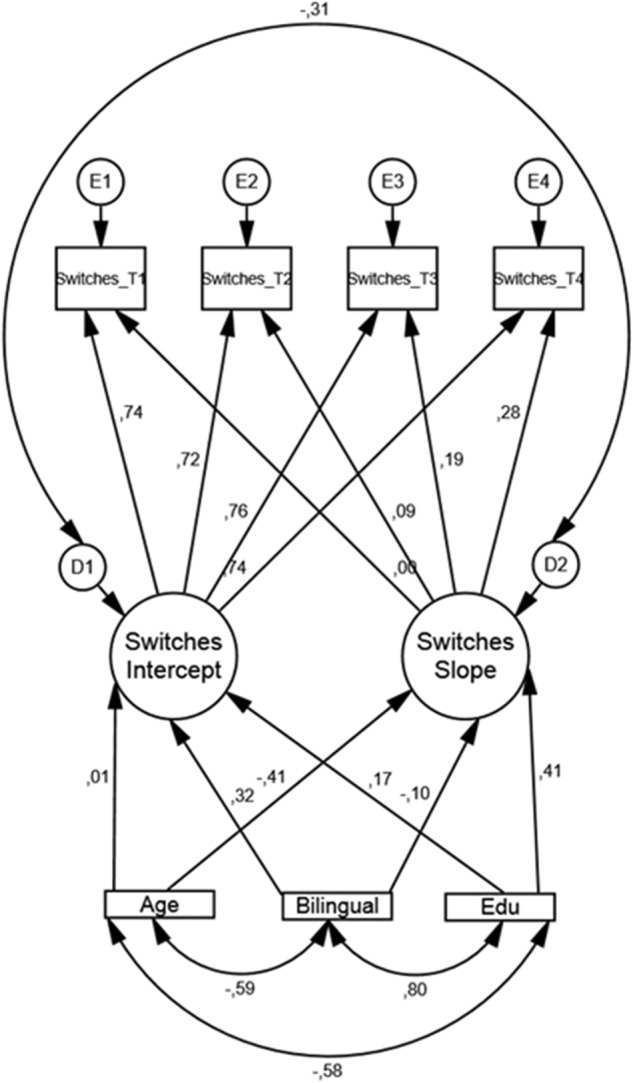
Conditional LGM for switching performance with bilingualism, age, and education as covariates along with standardized regression weights. Edu = years of formal schooling, Bilingual = (coded as 1 = yes and 0 = no).

Results from the conditional LGM, modeling switches (see Figure 2), are displayed in Table 5.

**Table 5:** Estimates from the Conditional LGM modeling switches using biligualism, age, and education as covarietes.

	Switches		
**Intercepts**	**Estimate**	***SE***	***P***

I (Intercept)	5.647	2.087	0.007
S (Slope)	0.093	0.015	0.535
**Residual variances**			
I (Intercept)	6.028	1.223	0.000
S (Slope)	0.003	0.008	0.742
Covariance of I and S	–0.040	0.830	0.633
**Covariate regressions**			
I on Bilingual	1.91	0.870	0.028
I on Age	0.003	0.032	0.339
I on Education	0.100	0.085	0.240
S on Bilingual	–0.015	0.630	0.810
S on Age	–0.003	(.002)	0.182
S on Education	0.006	(.006)	0.324

*I, intercept; S, slope*.

The fit indices indicated good model fit: CFI = 0.992, RMSEA = 0.046, and Chi-square/df = 1.423. In accordance with the unconditional LGM, only the residual variance was significant. That is, when regressing the covariates on intercept and slope there is still a non-trivial variation in baseline performance between participants. More importantly, of the three covariates, only Bilinguals (or monolinguals) were significantly associated with the intercept, that is, bilinguals had a higher performance score at baseline compared to monolinguals (β = 1.914, *SE* = 0.872, *p* = 0.028). Note that there are no significant associations between monolinguals or bilinguals regarding the rate of change over the four time points.

**FIGURE 3:**
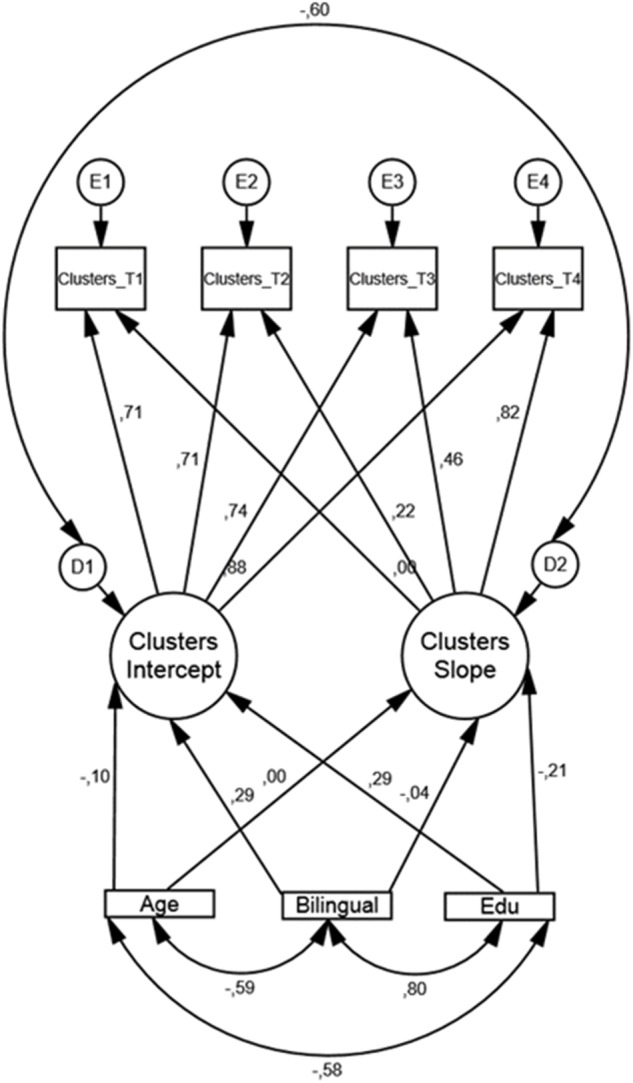
Conditional LGM for number of clusters with bilingualism, age, and education as covariates along with standardized regression weights. Edu = years of formal schooling, Bilingual = (coded as 1 = yes and 0 = no).

Results from the conditional LGM, modeling number of clusters (see Figure 3) are displayed in Table 6.

**Table 6:** Estimates from the Conditional LGM modeling clusters using biligualism, age, and education as covarietes.

	Clusters		
**Intercepts**	**Estimate**	***SE***	***P***

I (Intercept)	1.570	0.765	0.040
S (Slope)	0.020	0.069	0.772
**Residual variances**			
I (Intercept)	0.648	0.180	0.000
S (Slope)	0.004	0.002	0.017
Covariance of I and S	–0.030	0.014	0.040
**Covariate regressions**			
I on Bilingual	0.646	0.319	0.043
I on Age	–0.011	0.012	0.372
I on Education	0.063	0.031	0.045
S on Bilingual	–0.005	0.029	0.866
S on Age	0.000	0.001	0.986
S on Education	–0.003	0.003	0.304

*I, intercept; S, slope*.

The fit indices indicated good model fit: CFI = 0.979, RMSEA = 0.065, and Chi-square/df = 1.837. In accordance with the unconditional LGM, only the residual variance in both intercept and slope was significant. That is, when regressing the covariates on intercept and slope there is still a non-trivial variation in baseline performance and in rate of change between participants. Of the three covariates bilingualism was significantly associated (β = 0.646, *SE* = 0.319, *p* = 0.043) with the intercept. That is, on average, a bilingual participant had a higher performance score at baseline compared to a monolingual participant. Additionally, education was significantly associated (β = 0.063, *SE* = 0.031, *p* = 0.045) with higher performance score at baseline. Note that there are no significant associations between monolinguals and bilinguals or none of the other covariates regarding the rate of change over the four time points.

**FIGURE 4:**
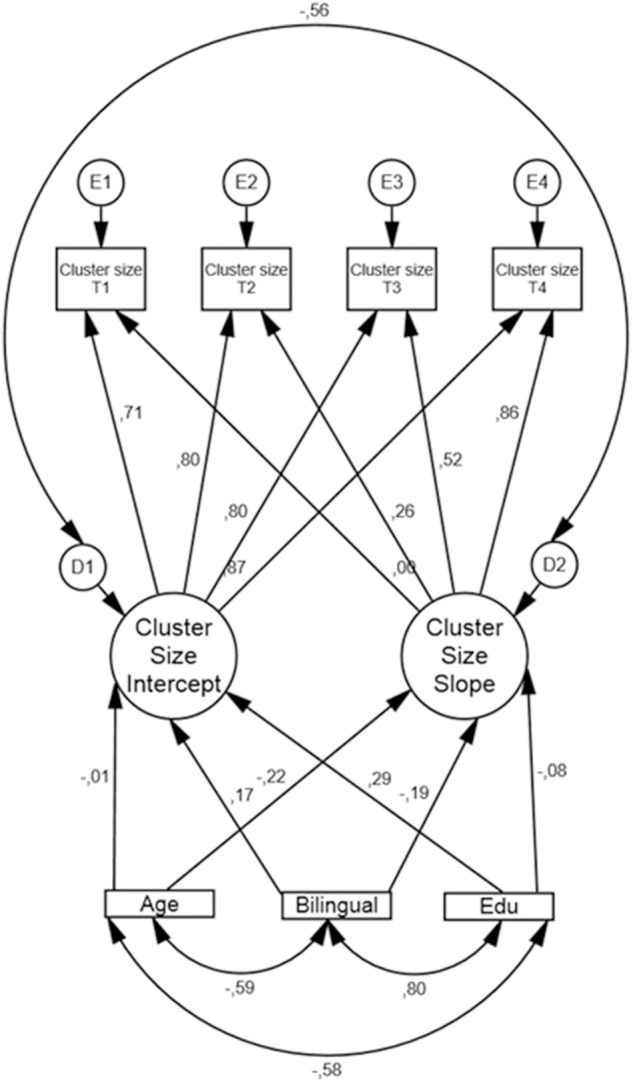
Conditional LGM of cluster size with bilingualism, age, and education as covariates along with standardized regression weights. Edu = years of formal schooling, Bilingual = (coded as 1 = yes and 0 = no).

Results from the conditional LGM, modeling cluster size (see Figure 4) are displayed in Table 7.

**Table 7:** stimates from the Conditional LGM modeling cluster size using biligualism, age, and education as covarietes.

	Cluster size		
**Intercepts**	**Estimate**	***SE***	***P***

I (Intercept)	1.561	1.616	0.334
S (Slope)	0.186	0.137	0.174
**Residual variances**			
I (Intercept)	3.494	0.723	0.001
S (Slope)	0.018	0.006	0.003
Covariance of I and S	–0.138	0.055	0.012
**Covariate regressions**			
I on Bilingual	0.771	0.675	0.254
I on Age	–0.001	0.025	0.960
I on Education	0.132	0.066	0.045
S on Bilingual	–0.058	0.057	0.310
S on Age	–0.003	0.002	0.117
S on Education	–0.002	0.006	0.681

*I, intercept; S, slope.*

The fit indices indicated good model fit: CFI = 0.995, RMSEA = 0.032, and Chi-square/df = 1.197. The residual variance in both intercept and slope was significant. That is, when regressing the covariates on intercept and slope there is still non-trivial variation in baseline performance and in rate of change between participants. Bilingualism was not significantly associated with the intercept (β = 0.771, *SE* = 0.675, *p* = 0.254), nor the slope (β = -0.058, *SE* = 0.057, *p* = 0.310). Additionally, more years of formal schooling were significantly (β = 0.132, *SE* = 0.066, *p* = 0.045) associated with higher performance score at baseline (intercept).

In summary: There was no time-related change (the slope) measured over 15 years in switching performance (i.e., number of switches between phonemic clusters of words). However, there were significant individual differences at baseline (the intercept). When slope and intercept were regressed on age, education, and bilinguals/monolinguals, the only association was between bilingualism and the intercept, that is, bilingual participants made more switches between phonemic clusters at baseline compared to monolingual participants (see Figure 2 and Table 5).

Regarding number of clusters, there was no overall time-related change but there was significant individual variance in the rate of change. However, none of the covariates used in our model managed to show significant association with this variation in slope. When intercept is regressed on the three covariates, both bilingualism and education were significantly and positively associated with baseline performance (see Figure 3 and Table 6).

For cluster size, there was an overall negative time-related change accompanied by significant individual variation in rate of change. None of the covariates used were associated with this variation. In terms of baseline performance only education showed a significant association with intercept (see Figure 4 and Table 7).

### Additional Analyses

Adding Block design and MMSE as covariates when modeling switching performance (model fit: CFI = 0.993, RMSEA = 0.039, and Chi-square/df = 1.304) showed that bilingualism was still significantly associated with the intercept (β = 1.794, *SE* = 0.842, *p* = 0.033). MMSE (β = 0.255, *SE* = 0.165, *p* = 0.006) and Block design (β = 0.064, *SE* = 0.027, *p* = 0.020) were both significantly associated with the intercept.

Regarding number of clusters (model fit: CFI = 0.982, RMSEA = 0.059, and Chi-square/df = 1.578) bilingualism remained significantly associated with the intercept (β = 0.633, *SE* = 0.314, *p* = 0.044). The same holds for MMSE (β = 0.152, *SE* = 0.061, *p* = 0.014), but not for Block design (β = 0.012, *SE* = 0.010, *p* = 0.252). When modeling cluster size, none of the covariates showed significant associations with the intercept nor the slope.

Further, we matched the participants on education (years), age, and sex (in that order). Using a Propensity Score matching procedure that comes with SPSS 23. We applied sampling without replacement, randomized case order when drawing matches, and a match tolerance (or distance) of 0.1. It resulted in very few participants in each group (*n* = 11). Mixed ANOVAs showed the same basic results as in our main analyses using LGM: No main effect of time, no interaction, but a main effect of language-group in that bilinguals perform better.

## Discussion

Verbal fluency is often used as a measure of executive control (e.g., Henry and Crawford, 2004; Fitzpatrick et al., 2013) and is underpinned by the executive processes of switching and clustering (e.g., Troyer et al., 1997). Generally, it is assumed that bilinguals possess greater executive control than monolinguals. This is hypothesized to emerge from the greater demands posed on inhibition, monitoring and switching between languages (Green, 1998). For example, when one language is in use, the other language remains active and requires to be inhibited (Green, 1998; Kroll et al., 2012). The lexical competition that results between languages in language production tasks is assumed to produce a bilingual disadvantage in lexical access (Luo et al., 2010). This disadvantage is related to the very mechanism that produces the bilingual advantage in executive control on non-verbal tasks or tasks that involve less cross-language interference: the practice that bilinguals continually experience in language control generalizes to tasks tapping cognitive or executive control that do not have a linguistic component (Bialystok et al., 2009).

However, there is mixed evidence for the proposed bilingual advantages in relation to executive processing (Bialystok et al., 2008a,b, 2014; Costa et al., 2009; Tao et al., 2011, 2015; Kousaie and Phillips, 2012; Paap and Greenberg, 2013; Duñabeitia et al., 2014; Valian, 2015). Notwithstanding this current discrepancy in findings, the results we report here demonstrate a clear bilingual advantage in the context of phonemic fluency (Ljungberg et al., 2013) which our new analysis demonstrates can be attributed to superior use of both clustering and switching executive processes among bilinguals as compared with monolinguals. While participants in the Betula study did not undertake the classic version of semantic fluency (and hence comparison between other studies of semantic fluency and our study here of phonemic fluency should be treated with caution) the results of the study are consistent with the notion that executive processing is more important for efficient performance in the context of phonemic as compared with semantic fluency and more classic tasks of lexical access (e.g., picture naming). In the current study, our re-analysis of phonemic fluency data from the Betula study, showed that bilinguals had superior clustering and switching at baseline (wherein participants were 35–65 years) and throughout the three further time-points of the study. Since switching and clustering in phonemic fluency are purportedly driven by executive function (e.g., Troyer et al., 1997, 1998), these findings are consistent with the view that superior executive processes underpin the bilingual advantage in phonemic fluency. Moreover, the results of the current study are consistent with previous research that has found that bilinguals outperform monolinguals in tests of phonemic fluency, with the reverse being more typical for semantic fluency (Rosselli et al., 2000; Gollan et al., 2002; Portocarrero et al., 2007).

Previous studies have suggested that bilingual advantages might be due to differences in vocabulary size with monolinguals having superior vocabularies to bilinguals. However, Luo et al. (2010) found an advantage in letter fluency for bilinguals with high vocabulary size compared to monolinguals and bilinguals low in vocabulary size. In the present study our bilinguals had larger vocabulary size than their monolingual counterparts and thus our conclusion that the bilingual advantage reflects superior executive functioning for bilinguals against monolinguals, coheres with the conclusion reached by Luo et al. (2010). On the face of it, it would seem sensible to control for vocabulary size while focusing on the differences in executive processes between bilingual and monolingual participants. However, we consider that this would be an ill-founded endeavor due to the overlap between the SRB vocabulary measure and the outcome measure – phonemic fluency (see e.g., Shao et al., 2014; Gordon et al., 2018), which itself has been used to measure vocabulary size.

However, it is important to note that the longitudinally demonstrated advantage of bilinguals over monolinguals for switching and clustering held when years of education, a significant predictor of performance on the phonemic fluency task (Tallberg et al., 2008; see also Troyer, 2000) and associated with cognitive reserve (Barnes et al., 2004; Scarmeas et al., 2006) were controlled. Furthermore, the results also held when controlling for the participants global cognitive status and visuospatial ability, the later correlates strongly with measures of general intelligence (Ryan et al., 1990). Additionally, a matched-samples procedure (matched on age, education, and sex) was used. Despite the fact that the matching procedure resulted in very few participants in each group (*n* = 11) the basic results from the LGM modeling, and thus the conclusions reached, were the same.

While we found a reliable effect of bilingualism on switching and clustering that are thought to be underpinned by executive function in phonemic fluency, other studies have failed to find a direct association between bilingualism and executive function (e.g., Paap and Greenberg, 2013). One possible reason why we found an association in the current study compared to other studies (e.g., Paap and Greenberg, 2013) is that we used older adults and had a monolingual group that were not familiar with any other languages. Cognitive control abilities for example may be at ceiling level for younger adults thereby potentially masking bilingual advantage/disadvantage effects across studies (Tao et al., 2015). Older adults are a more heterogeneous population than younger adults in terms of executive function and therefore the opportunity for observing an association is greater (e.g., Ardila, 2007). However, other factors such as bilingual balance may also be driving the effect of bilingualism on phonemic fluency in the current study. Given the available data, we were not able to compare balanced with unbalanced individuals. There is suggestion (e.g., Gollan et al., 2002; Bialystok et al., 2006; but see Duñabeitia et al., 2014) that the bilingual advantage in phonemic fluency, and bilingual disadvantage in semantic fluency, emerges from balanced, as compared to unbalanced, bilingualism. The latter occurring due to cross-language interference which is more pronounced for category than for phonemic fluency. However, it has also been argued that the production of the dominant language is invulnerable to competition between languages (Gollan et al., 2008), particularly in balanced bilinguals (Costa and Santesteban, 2004). Therefore further efforts are required to examine the relationship between balanced and unbalanced bilingualism on the bilingual advantage as measured by phonemic fluency.

A further point of interest in our study is the potential association between age and verbal fluency. Several studies report decreasing verbal fluency as a function of age (Crossley et al., 1997; Troyer et al., 1997; Troyer, 2000). While semantic fluency often demonstrates age-related declines in fluency (Crossley et al., 1997; Troyer et al., 1997; Troyer, 2000), the results are indifferent in relation to phonemic fluency with some reporting stability across the age ranges tested (e.g., Crossley et al., 1997; Troyer et al., 1997; Ljungberg et al., 2013) while others show an age-related decline in the number of words produced (e.g., Ostrosky-Solís et al., 1999; Brickman et al., 2005; Rodríguez-Aranda and Martinussen, 2006). It has been proposed that differences manifest through an age-related decline in the efficiency of executive function, rather than accessibility of semantic knowledge for retrieval. For example, in their cross-sectional study, Troyer et al. (1997) found that younger adults’ fluency performance differed from adults due to lower switching scores despite comparable clustering in semantic fluency (see also Sauzéon et al., 2011). In the context of phonemic fluency, older adults generated larger clusters than younger adults (Troyer et al., 1997). However, no age-related differences in cluster size, nor switching were observed in our study.

Very few studies have examined whether the bilingual advantage in executive function persists in older age. However, Bialystok et al. (2004; see also Rosselli et al., 2000) showed that the advantage of bilinguals over monolinguals in executive control remained in older adulthood with the advantage being accentuated for the older group of bilinguals. Since cognitive control and lexical access tend to decrease with older age (Bialystok et al., 2004; McDowd et al., 2011; Röer et al., 2015) it may be speculated that bilingualism aids the compensation of age-related decline in particular cognitive processes. However, while the bilingual advantage may become more pronounced in older age, the bilingual disadvantage in relation to naming difficulties and lexical access is observed in older adults and shows a similar decline to monolingual participants (Gollan et al., 2007; Bialystok et al., 2008a). In contrast to the study by Bialystok et al. (2004) we observed a bilingual advantage at baseline for switching and clustering but this advantage did not become accentuated for later time-points. Rather, the difference was stable over time, thus bilinguals could have an advantage over monolinguals since they enter old age with better cognitive ability than monolinguals. It should be noted though that at the end of the study, most of our bilingual participants were still under the age of 65, whereas the old participants in the Bialystok et al. (2004) study had passed retirement age and had a mean age over 70 years. The failure to find an accentuation of differences in executive processes between monolinguals and bilinguals over time may be due to the general age-invariant property of phonemic, as compared with semantic, fluency. Troyer et al. (1997) for example, found age-related decline in semantic fluency, but not phonemic fluency. In this regard we cannot speculate on the idea that the consistent use of more than one language can stave off some of the symptoms of normal cognitive aging (Kavé et al., 2008) and protect against the onset of the cognitive symptoms of dementia (Bialystok et al., 2007; Craik et al., 2010).

While we have associated clustering and switching with executive function, we have not yet speculated as to which executive function (inhibition, shifting, updating; Miyake et al., 2000) they are (more strongly) linked. In our view switching appears to be most straightforwardly related to the executive function of shifting. This would cohere with the well-cited rationale that executive functions such as shifting are improved due to the bilingual requirement to shift back and forth rapidly between two languages. Several reports suggest bilinguals have superior shifting ability. For example, in a study requiring switching between judgments of color (red or green) and shape (circle or triangle), bilinguals were faster on trials requiring switching between the judgments as compared to no-switch trials, thereby demonstrating smaller switch costs (Prior and MacWhinney, 2010). Within the context of such tasks neuroimaging studies have demonstrated activation of non-linguistic monitoring and inhibitory processes in monolinguals whereas bilingual language control areas are activated in bilinguals during the same task (Garbin et al., 2010). This suggests that areas used for language shifting are co-opted by bilinguals to perform non-linguistic tasks that also require switching. However, while the executive function of shifting would fit well with switching flexibly between retrieval of one cluster to another in the context of verbal fluency, it is not clear how the shifting is associated with the clustering process. This process might be underpinned by another executive function. Consistent with this reasoning, fluency is likely underpinned by many executive processes. Indeed Shao et al. (2014) report that fluency is associated with the executive process of updating and that this process is involved in keeping track of words that they have already produced to avoid repetition (e.g., perseverative errors). Future studies should attempt to address the association of switching and clustering with independent measures of executive control such as those tapped by the tasks used by Miyake et al. (2000), assuming that the classification of tasks according to which executive function they purportedly tap, is correct which is currently uncertain (Sörman et al., 2019).

While the dominant explanation for a bilingual advantage in the context of phonemic fluency has been attributed to bilingual advantages in executive processing (Rosselli et al., 2000; Gollan et al., 2002; Portocarrero et al., 2007) an alternative account, based on lifelong learning (Ramscar et al., 2014, 2017) is gaining currency as an explanation of age-related decline and bilingual differences in cognitive performance on verbal cognitive tasks. This approach proposes that older adults, and monolinguals, have increased linguistic exposure and learning resulting in the accumulation of knowledge. This increased language exposure and learning results in an information processing cost: choosing or recalling items is rendered more difficult due to the presence of numerous other items within the cognitive system. In the case of semantic fluency—e.g., recalling animal names—production of exemplars is impeded as more animal names are learnt over time. However, in the case of phonemic fluency, the potential impedance to the retrieval of items produced by increasing vocabulary acquisition, could be offset by a reduction in the retrieval of cues that would interfere with the production of valid items (Ramscar et al., 2014, p. 31). This could explain why age-related decreases in phonemic, relative to semantic fluency, are more rarely observed (see e.g., Gordon et al., 2018).

How might this “cost of learning” perspective (Ramscar et al., 2014, 2017) explain the bilingual advantage in phonemic fluency we report here? One possible explanation is that monolinguals, due to their greater linguistic exposure, have learnt and acquired many more words, word forms, and associations between these, as compared with bilinguals. As previously mentioned, phonemic fluency requires participants to suppress the dominant use of words according to their meaning (Perret, 1974), possibly by inhibiting semantic associations between words. According to the cost of learning view (Ramscar et al., 2014, 2017), the consequence of this inhibition of semantic associations, as evident in poorer phonemic fluency performance, would be greater for monolinguals whom possess more knowledge of semantic associations between words than do bilinguals. Therefore, in line with the current findings, bilinguals as compared with monolinguals would face smaller costs of having to inhibit semantic associations in phonemic fluency, thereby allowing them to switch between phonemic categories with greater ease. Further work using verbal fluency tasks is required to fully examine the credentials of the cost of learning view (Ramscar et al., 2014, 2017) against the executive function view (e.g., Troyer et al., 1998; Friesen et al., 2015) in explaining bilingual advantages/disadvantages.

Verbal fluency tasks have many purposes. They have been used to assess cognitive impairment in Alzheimer’s (Laws et al., 2010; Zhao et al., 2013), Parkinson’s (Pettit et al., 2013) and other neurodegenerative diseases. Moreover, different brain regions are activated in phonemic than semantic fluency tasks (e.g., Schwartz and Baldo, 2001; Grogan et al., 2009; Quinn et al., 2012; Katzev et al., 2013). Brain regions associated with better phonemic fluency have also been shown to be active during bilingual language switching (Luk et al., 2012). This harmonizes well with the findings of the current study that the bilingual advantage in phonemic fluency is underpinned by executive function processes (switching and clustering): processes that are arguably not as central to other forms of verbal retrieval that are more conceptually driven and susceptible to semantic interference between languages (e.g., semantic fluency and picture-naming).

## Ethics Statement

This study was approved by the Ethics Review Board, Umeå University. All subjects gave written informed consent in accordance with the Declaration of Helsinki.

## Author Contributions

JM, PH, DS, and JL developed the research questions and wrote the introduction, methods, results, and the conclusion sections. JM and PH performed the formal analyses. All authors contributed equally.

## Conflict of Interest Statement

The authors declare that the research was conducted in the absence of any commercial or financial relationships that could be construed as a potential conflict of interest.
