# Supplementary material for: Executive Processes Underpin the Bilingual Advantage on Phonemic Fluency: Evidence From Analyses of Switching and Clustering
Source: Front Psychol. 2019 Jun 12;10:1355. doi: 10.3389/fpsyg.2019.01355 (PMC6581746; doi:10.3389/fpsyg.2019.01355)
Supplement: Supplementary file 1 [file Table_1.DOCX]

**Supplementary material**

Phonemic fluency performance in terms of number of words

Results from modeling number of words (see **Figure A1**), are displayed in **Table A1**. In accordance with Ljungberg et al. (2013) Bilinguals (or monolinguals) was significantly associated with the intercept, that is, bilinguals had a higher performance score at baseline compared to monolinguals (β = 2.617, *S.E.* = .993, *p* =.008). Note that there are no significant association between monolingual or bilingual regarding the rate of change over the four time points (see **Table A1**) which resembles the non-significant interaction between group and time in Ljungberg et al. (2013).

**
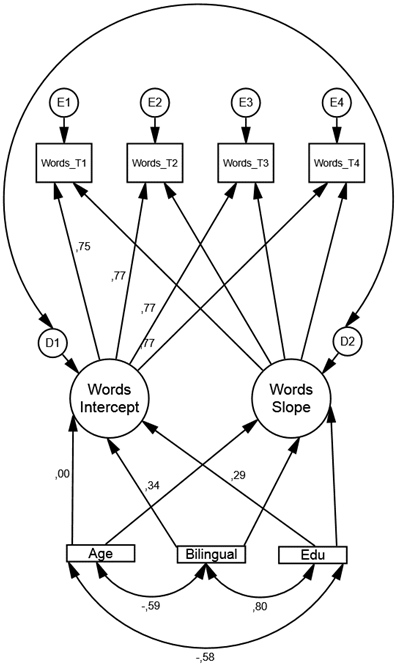
**

**Figure A1** Conditional LGM for number of words with bilingualism, age, and education as covariates along with standardized regression weights. Edu = years of formal schooling, Bilingual = (coded as 1= yes and 0 = no).

**Table A1** Estimates from the Conditional LGM modeling number of words using biligualism, age, and education as covarietes.

|  |  | **Words** |  |  |
| --- | --- | --- | --- | --- |
| **Intercepts** |  | **Estimate** | ***S.E.*** | ***P*** |
| I (Intercept) |  | 8.149 | 2.378 | .001 |
| S (Slope) |  | .289 | .162 | .074 |
| Residual variances |  |  |  |  |
| I (Intercept) |  | 7.580 | 1.543 | .001 |
| S (Slope) |  | -.004 | .010 | .658 |
|  |  |  |  |  |
| Covariance of I and S |  | .047 | .098 | .632 |
|  |  |  |  |  |
| Covariate regressions |  |  |  |  |
| I on Bilingual |  | 2.617 | .993 | .008 |
| I on Age |  | .000 | .037 | .994 |
| I on Education |  | .220 | .097 | .024 |
|  |  |  |  |  |
| S on Bilingual |  | -.091 | 0.993 | .180 |
| S on Age |  | -.007 | .002 | .008 |
| S on Education |  | .005 | .007 | .477 |
|  |  |  |  |  |
